# Successful Long-Term Treatment of Pediatric Relapsing Idiopathic Optic Neuritis with Mycophenolate Mofetil

**DOI:** 10.3390/neurolint17030044

**Published:** 2025-03-18

**Authors:** Shuhei Fujino, Keiji Akamine, Eiichiro Noda, Sahoko Miyama

**Affiliations:** 1 Department of Neurology, Tokyo Metropolitan Children’s Medical Center, Tokyo 183-8561, Japan; sahoko_miyama@tmhp.jp; 2 Department of Nephrology, Tokyo Metropolitan Children’s Medical Center, Tokyo 183-8561, Japan; keiji_akamine@tmhp.jp; 3 Department of Ophthalmology, Tokyo Metropolitan Children’s Medical Center, Tokyo 183-8561, Japan; eiichirou_noda@tmhp.jp

**Keywords:** pediatric optic neuritis, relapsing idiopathic optic neuritis, corticosteroids, mycophenolate mofetil, neuromyelitis optica spectrum disorder, immunosuppressives, long-term treatment

## Abstract

**Background:** Pediatric optic neuritis (ON) is a rare but severe condition characterized by acute visual impairment, with 3–5% of relapsing cases lacking identifiable markers for associated conditions, such as neuromyelitis optica spectrum disorder (NMOSD) or multiple sclerosis (MS); these cases are thus classified as relapsing idiopathic optic neuritis (RION). Corticosteroids are typically used for acute management; however, their prolonged use in children poses significant risks, including central obesity, hypertension, and growth impairment, underscoring the need for nonsteroidal, long-term treatment options. Current strategies for preventing recurrence in pediatric RION are limited due to a lack of data on immunosuppressive efficacy and safety. Given its rarity and the challenges of long-term immunosuppression in children, identifying optimal therapeutic approaches remains critical. **Case Presentation:** We report a case of a six-year-old girl with RION, who was initially treated with intravenous methylprednisolone (IVMP) and prednisolone (PSL) tapering, and who experienced recurrence eight months post-treatment. Additional corticosteroids and intravenous immunoglobulin (IVIg) were administered during relapse, but, due to adverse effects, treatment was transitioned to mycophenolate mofetil (MMF), enabling early PSL tapering. **Conclusions:** With MMF, the patient maintained stable vision and achieved a five-year recurrence-free period without notable side effects. In conclusion, this case suggests MMF’s efficacy as a long-term management option for pediatric RION, potentially reducing corticosteroid-related risks.

## 1. Introduction

Pediatric optic neuritis (ON) is a rare disease characterized by subacute severe visual impairment, often bilateral, with an estimated annual incidence of less than 0.2–1.7 per 100,000 person-years [1,2,3]. Pediatric ON may present as a manifestation of diseases such as multiple sclerosis (MS), neuromyelitis optica spectrum disorder (NMOSD), acute disseminated encephalomyelitis (ADEM), and other systemic autoimmune diseases, all of which are reported as significant risk factors for recurrence [4,5,6]. Predictors of MS include white matter abnormalities detected on an MRI, the presence of oligoclonal bands (OCB), and elevated myelin basic protein (MBP) in the cerebrospinal fluid (CSF) [1,4,6]. In contrast, NMOSD and ADEM are associated with specific autoantibodies, such as anti-aquaporin-4 antibody (AQP4-Ab) and anti-myelin oligodendrocyte glycoprotein antibody (MOG-Ab), as well as distinct MRI findings [1,4,6,7]. In most cases of recurrent ON, an underlying demyelinating disease is identified; however, in 3–5% of cases where the workup for comorbid demyelinating diseases is negative, the condition is diagnosed as recurrent idiopathic optic neuritis (RION) [1,6,8,9].

The acute treatment of ON involves a regimen combining intravenous methylprednisolone pulse therapy (IVMP) followed by oral prednisolone (PSL) tapering [1,6,10]. For steroid-refractory cases, intravenous immunoglobulin therapy (IVIg) or plasmapheresis therapy are considered complementary treatments during the acute phase [1,6,10]. This approach is based on findings from the North American Optic Neuritis Treatment Trial (ONTT), which focused on adult ON cases [6,10]. In pediatric cases of ON, oral prednisolone (PSL) tapering is recommended for at least four to six weeks, a duration longer than that suggested for adults, to reduce the risk of relapse [6]. However, long-term PSL therapy presents significant challenges due to steroid-induced adverse effects [6]. The rarity of pediatric ON further limits the availability of prospective data on the efficacy of immunosuppressive agents for recurrence prevention, leaving no established, evidence-based long-term strategies [6]. Given these challenges, identifying optimal therapeutic approaches remains essential for improving management of this rare condition. We herein report the case of a six-year-old female patient with RION who relapsed after the discontinuation of corticosteroid therapy, in whom mycophenolate mofetil (MMF) was effective at preventing further recurrence.

## 2. Case Presentation

### 2.1. Clinical Course of the First Onset of ON

A six-year-old girl presented to our clinic with bilateral visual impairment that had persisted for two weeks. She had no significant past medical or family history, no symptoms of infection, and no history of recent vaccinations within the last few months. She lived in an urban area and had no history of fever, fatigue, or rash in the past few months, no tick bites, no history of pet ownership, and no previous bites or scratches. Additionally, she had no history of sexual contact. Six months prior, her visual acuity (VA) had been documented as 20/20 or better bilaterally during a routine school health checkup using the Landolt ring chart.

**Figure 1:**
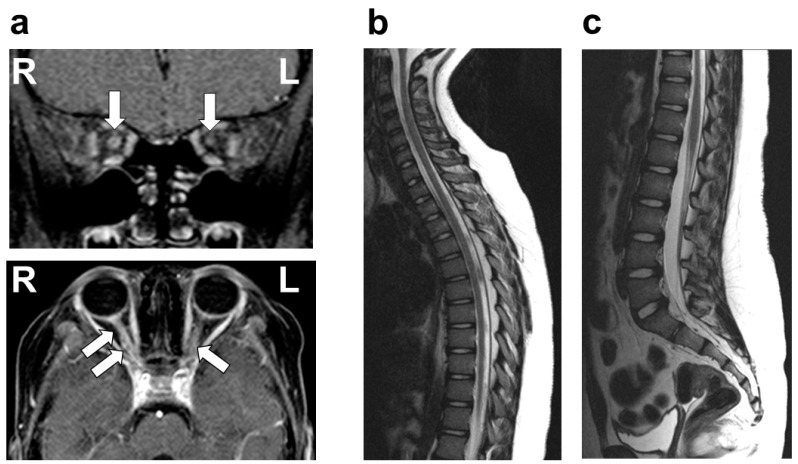
Brain and spine MRI after the first attack of optic neuritis. (**a**) Brain gadolinium-enhanced MRI (T1-weighted image) showing significant enhancement of the bilateral optic nerve with right-sided predominance (white arrow). The upper and lower panels represent the coronal and horizontal sections, respectively. (**b**,**c**) Thoracic spine MRI (**b**), and lumbar and sacral spine MRI (**c**), showing no abnormalities in signal intensity (sagittal T2-weighted image).

On physical examination, she was afebrile, with no cervical lymphadenopathy. Her pupils measured 6 mm on the right and 4 mm on the left, both showing sluggish light reflexes bilaterally. No other neurological abnormalities were identified, with no evidence of the involvement of cranial nerves other than the optic nerve, including the trigeminal and facial nerves. VA testing revealed no light perception in the right eye, while the left eye demonstrated a VA of 20/30. A fundus examination showed bilateral optic disc redness and inflammatory edema. There was no evidence of involvement beyond the optic nerve, including the uvea, sclera, cornea, conjunctiva, or chorioretina. Optical coherence tomography (OCT) demonstrated pronounced bilateral optic disc swelling, predominantly affecting the right eye. Flash-visual evoked potential (Flash-VEP) revealed prolonged P100 latency bilaterally. A contrast-enhanced MRI of the head revealed significant enhancement of the bilateral optic nerves, predominantly on the left side (Figure 1). No other abnormalities were observed in the brain or spinal cord MRIs (Figure 1). A chest X-ray revealed no pulmonary nodular opacities or hilar lymphadenopathy. The laboratory investigations, including blood counts, biochemical parameters, and coagulation profiles, were unremarkable. Serum tests for antinuclear antibody, dsDNA antibody, anti-SS-A/SS-B antibody, anti-MPO-ANCA antibody, and anti-PR3-ANCA antibody were all negative. The CSF analysis showed normal cell counts, glucose, and protein levels. Further CSF studies revealed that AQP4-Ab (measured via an enzyme-linked immunosorbent assay (ELISA)), MOG-Ab (measured using a live cell-based assay (live CBA)), OCB, and MBP were all negative. Based on these findings, the patient was diagnosed with idiopathic ON.

Treatment was initiated on day 2 with IVMP at a dose of 1 g/day for 3–5 days per course, repeated after a one-week interval. Following IVMP, oral PSL therapy was started at 1 mg/kg/day and tapered over three months. VA showed improvement approximately one week after the initial IVMP course. By day 23, VA had improved to 10/20 in the right eye and 20/20 in the left eye. On day 23, OCT demonstrated improvement in bilateral optic disc swelling, and flash-VEP on the same day showed normalization of P100 latency in both eyes. Further recovery of VA was observed, reaching 20/20 in the right eye and 20/13 in the left eye two months after the initiation of treatment.

During the three-month oral PSL tapering period following the IVMP therapy, the patient developed hypertension, increased appetite, and central obesity. These adverse effects were resolved following the tapering and discontinuation of PSL.

### 2.2. Clinical Course After Recurrence

Eleven months after the initial onset (eight months following the cessation of steroid therapy), the patient experienced recurrent bilateral visual impairment, with a VA of 20/2000 in the right eye and 20/30 in the left eye. A fundus examination showed bilateral papilledema. As was the case at the initial presentation, the patient remained afebrile, with no ocular findings beyond the optic nerve, no cranial nerve abnormalities, no systemic neurological deficits, and no lymphadenopathy. The chest X-ray findings also remained within the normal limits. The brain gadolinium-enhanced MRI showed enhancement of both optic nerves, consistent with the initial findings. No other abnormalities were observed in the brain or spinal cord MRI. Serum autoantibody tests, including AQP4-Ab (measured via ELISA) and MOG-Ab (measured via live CBA), as well as CSF analyses for OCB and MBP, were all negative. Based on the absence of findings suggestive of MS or NMOSD and the recurrence after steroid discontinuation, a diagnosis of RION was made.

A total of three courses of IVMP therapy (1 g/day for three days per course) and IVIg therapy (2 g/kg over five days) were administered. Following the acute phase, oral PSL at 1 mg/kg/day was initiated.

Given the condition’s recurrence after a three-month corticosteroid tapering period, it was deemed necessary to implement immunosuppressive therapy for a longer duration. However, central obesity and secondary hypertension, side effects previously observed during the first episode, emerged again as challenges with the corticosteroid therapy. To mitigate these issues, the oral PSL dose was reduced as early as possible, and the patient was transitioned to alternative immunosuppressive agents.

At 1.5 months after the second onset, azathioprine (1 mg/kg/day) was initiated alongside the tapering of oral PSL. However, azathioprine was discontinued two months later due to elevated liver enzyme levels and was subsequently replaced with MMF at a dose of 1200 mg/m^2^/day. Oral PSL was gradually tapered at a rate of 0.25 mg/kg/day every two weeks. Four months after the recurrence, oral PSL was transitioned to alternate-day administration and was subsequently discontinued 15 months post-recurrence. During the oral PSL treatment period, side effects, including central obesity and hypertension, were observed, as had been noted during the initial episode. However, following the initiation of alternate-day PSL dosing, these adverse effects improved significantly and came under control. VA ultimately recovered to over 20/20 bilaterally within one year after the second onset. During the five-year follow-up period, the patient underwent ophthalmologic and neurological examinations every three months, with no abnormalities observed in visual acuity, visual field, or color vision. MMF effectively maintained a recurrence-free state without any reported adverse effects.

## 3. Discussion

This case report highlights the potential efficacy of MMF in preventing recurrence in pediatric RION following corticosteroid discontinuation. In pediatric RION, established guidelines regarding the selection of immunosuppressive agents and the optimal duration of their administration are currently lacking. According to the ONTT, the standard treatment for ON, which is primarily based on studies in adults, includes IVMP at 4–30 mg/kg/day for three to five days, followed by oral PSL tapering [6,10]. In pediatric cases, a longer PSL tapering period of at least four to six weeks is recommended compared to adults to reduce the risk of relapse [6,10]. For acute steroid-refractory cases, complementary interventions such as IVIg or plasmapheresis may be considered [1,6,10]. Prolonged corticosteroid therapy in pediatric patients, however, poses substantial challenges due to its well-documented adverse effects, including hypertension, central obesity, and, particularly in children, growth impairment [6,11]. In the present case, the patient suffered a relapse after three months of corticosteroid therapy, accompanied by notable side effects such as central obesity and hypertension. These findings highlight the necessity of a long-term immunosuppressive strategy to prevent recurrence while avoiding the complications associated with prolonged corticosteroid use. To address these challenges, azathioprine was initially introduced as an immunosuppressive therapy for relapse prevention. However, the patient developed drug-induced liver injury, necessitating the discontinuation of azathioprine. Subsequently, MMF was administered, leading to a recurrence-free period without significant adverse events. If the therapeutic effect of MMF is insufficient, alternative options such as regular IVIG administration or anti-CD20 agents such as rituximab should be considered [12]. These results suggest that MMF may be an effective and safer alternative to corticosteroids for managing pediatric RION.

In this case, a five-year long-term immunosuppressive therapy with MMF was administered for pediatric RION, resulting in a favorable outcome. Long-term management strategies for NMOSD and steroid-dependent recurrent ON in adults have demonstrated the efficacy of immunosuppressive agents, including MMF, azathioprine, methotrexate, and monoclonal antibodies such as rituximab, with or without corticosteroids [9,11]. In NMOSD, where immune-mediated mechanisms have been established and autoantibodies such as AQP4-Ab are identified, long-term immunosuppressive therapy is recommended for at least five years in treatment-resistant or recurrent cases to prevent further relapses [11]. For adult steroid-dependent recurrent ON, systematic reviews suggest that controlling disease activity may require treatment to be extended over a decade [13]. Accordingly, although neither NMOSD nor autoantibody positivity was identified in this patient, the recurrence observed several months after the initial attack suggested the necessity of long-term treatment strategies, lasting at least several years, aligned with NMOSD recurrence-prevention protocols. Consequently, a five-year course of MMF was implemented, ensuring sustained disease control and effective relapse prevention. This outcome supports the potential efficacy of MMF as a long-term therapeutic strategy for managing pediatric RION.

In cases of ON, particularly RION, the potential progression to MS or NMOSD should be carefully considered. Generally, ON is closely associated with MS and NMOSD, with reports indicating that approximately 10–40% of pediatric ON cases are later diagnosed with MS, and 1–5% progress to NMOSD [3,6,7,9,14]. Even in cases such as our case, where clinical or diagnostic findings indicative of MS or NMOSD are absent, it remains crucial to consider the possibility that ON may represent an early manifestation of these underlying conditions [3,6,7,9,14]. In our case, regular ophthalmologic and neurological evaluations were conducted to detect potential progression to MS, NMOSD, or other related conditions during the course of the chronic immunosuppressive therapy. Additionally, although extensive infectious screening was not performed in this case due to the absence of clinical signs suggestive of systemic infection and low epidemiological risk, it is essential to carefully assess systemic involvement to rule out infectious causes of optic neuritis, such as Bartonella [15], Lyme disease [16,17], and syphilis [18]. Moreover, while the long-term follow-up revealed no evidence of malignancy, recurrence or disease progression would prompt screening for paraneoplastic syndromes, particularly those associated with CRMP5/CV2 antibodies, which are among the more frequently observed causes of optic neuritis in paraneoplastic syndromes [19]. Glial fibrillary acidic protein (GFAP) antibody-associated disorder is another important differential diagnosis [20], as it can present with diverse neurological symptoms, including optic neuritis and optic disc edema. It is frequently associated with increased protein levels, elevated mononuclear cell counts, and positive OCB in the CSF [21,22]; however, these findings were absent in this case. Although the inflammation was confined to the optic nerve and no signs of uveitis were observed, and there was no characteristic hilar lymphadenopathy, sarcoidosis remains a potential cause of optic neuropathy [23,24]. The lack of comprehensive screening for these conditions is a limitation of this study, as they cannot be entirely excluded. However, the absence of related complications over more than five years of follow-up supports the diagnosis of idiopathic ON. Further relapses should prompt additional screening for these conditions. Patients with RION should be closely monitored for changes in visual acuity and other systemic or neurological findings throughout the course of chronic immunosuppressive therapy to detect potential progression to these or other related conditions.

## 4. Conclusions

This case demonstrates that MMF may serve as a valuable therapeutic option for preventing recurrence in pediatric RION cases that relapse following the discontinuation of steroids. Given the rarity of RION in children and the limited treatment options available, our findings provide important insights into its management. Careful monitoring for adverse effects is essential when selecting immunosuppressive agents for preventing recurrence, underscoring the importance of individualized treatment strategies. Further studies are warranted to assess the safety and long-term efficacy of immunosuppressive therapies in managing pediatric RION.

## Data Availability

The data that support the findings of this report are available from the corresponding author, S.F., upon reasonable request.
